# Residual Energy Estimation-Based MAC Protocol for Wireless Powered Sensor Networks

**DOI:** 10.3390/s21227617

**Published:** 2021-11-16

**Authors:** Sol-Bee Lee, Jung-Hyok Kwon, Eui-Jik Kim

**Affiliations:** School of Software, Hallym University, 1 Hallymdaehak-gil, Chuncheon 24252, Gangwon-do, Korea; thfqla3535@hallym.ac.kr (S.-B.L.); jhkwon@hallym.ac.kr (J.-H.K.)

**Keywords:** energy harvesting, Internet of Things, out-of-band approach, residual energy estimation, wireless power transfer, wireless powered sensor network

## Abstract

This paper presents a residual energy estimation-based medium access control (REE-MAC) protocol for wireless powered sensor networks (WPSNs) composed of a central coordinator and multiple sensor devices. REE-MAC aims to reduce overhead due to control messages for scheduling the energy harvesting operation of sensor devices and provide fairness for data transmission opportunities to sensor devices. REE-MAC uses two types of superframes that operate simultaneously in different frequency bands: the wireless energy transfer (WET) superframe and wireless information transfer (WIT) superframe. At the beginning of each superframe, the coordinator estimates the change in the residual energy of individual sensor devices caused by their energy consumption and energy harvesting during the previous superframe. It then determines the devices’ charging priorities, based on which it allocates dedicated power slots (DPSs) within the WET superframe. The simulation results demonstrated that REE-MAC exhibits superior performance for the harvested energy, average freezing time, and fairness to existing representative WPSN MAC protocols.

## 1. Introduction

Recent advances in radio frequency (RF)-based wireless energy transfer (WET) techniques enable battery-powered sensor devices to receive energy remotely without time and space constraints on ambient resources such as solar, thermal, wind, and vibration, enabling perpetual operations. Thus, wireless sensor networks (WSNs) with RF-based WET—wireless powered sensor networks (WPSNs)—are considered one of the most promising technologies for a sustainable Internet of Things [1,2,3,4,5,6,7,8,9]. In the WPSN, a power station wirelessly transfers energy to sensor devices that use the harvested energy to transmit their collected information to a fusion center [10,11,12]. The power station and fusion center may be included in one device or separated into different devices.

Although such WPSNs are expected to have a potentially infinite network life, they cannot always prevent short-term disconnections due to temporary energy shortages of some sensor devices. Such short-term disconnections cause an imbalance in transmission opportunities between sensor devices, resulting in an unfairness problem for WPSNs [13,14,15,16]. Moreover, in contrast to the traditional WSNs, in the WPSN, when sensor devices operate, wireless information transfer (WIT) and WET must be jointly considered. Consequently, it is necessary to design an appropriate medium access control (MAC) protocol for WPSNs.

Many studies have been conducted to design an efficient MAC protocol for WPSNs. In [17,18,19], the authors proposed a MAC protocol based on carrier-sense multiple access with collision avoidance (CSMA/CA) for WPSNs. Sensor devices access the channel competitively to conduct energy harvesting and data transmission; their channel access priorities are probabilistically differentiated by the backoff duration and inter-frame space (IFS), determined by the remaining energy. In [20,21,22], the authors proposed the time-division multiple access (TDMA)-based MAC protocol, in which a central coordinator allocates time resources for energy harvesting and data transmission considering the remaining energy of the sensor devices and the energy consumption required for data transmission. The sensor devices harvest energy in the dedicated time slots and transmit data.

Cho et al., used both TDMA and CSMA/CA methods to support energy harvesting and data transmission for two types of traffic patterns: periodic and non-periodic [23]. In [23], a coordinator allocates the dedicated TDMA time slots for energy harvesting and data transmission within a superframe to sensor devices that generate periodic traffic requiring transmission reliability on-demand. In contrast, energy harvesting and data transmission for non-periodic traffic are performed in the rest of the superframe using the CSMA/CA scheme. The studies in [17,18,19,20,21,22,23] commonly suffer from network performance degradation due to a lack of bandwidth resources from performing both WET and WIT operations within the same frequency band.

In [24,25,26], the authors used an out-of-band WET approach where sensor devices perform WET and WIT operations independently in different frequency bands, mitigating the effect of bandwidth limitation on WIT operation and improving network performance. However, their research suffers from high overhead due to the exchange of numerous control messages required to schedule WET and WIT operations in separate frequency bands.

Furthermore, the WET scheduling method used in [24,25,26] depends on simple criteria such as the distance to the sensor device and the energy required to transmit the data packet, increasing the difference in the residual energy between the sensor devices in the network. This difference causes an imbalance in transmission opportunities between sensor devices, resulting in an unfairness problem for network performance. This unfairness problem also applies to the in-band WET approach of [17,18,19,20,21,22,23].

This paper proposes a residual energy estimation-based MAC (REE-MAC) protocol, with two advantages for WPSNs composed of a central coordinator and multiple sensor devices. First, REE-MAC increases the residual energy of individual sensor devices by reducing overhead due to control messages for scheduling the energy harvesting operation of sensor devices. The coordinator numerically estimates the residual energy of individual sensor devices rather than exchanging numerous control messages. Second, REE-MAC establishes fairness among the data transmission opportunities for sensor devices. The coordinator allocates WET slots within the superframe to the sensor device by comprehensively considering the distance, harvested energy, and consumed energy for individual sensor devices. Accordingly, the residual energy of the sensor devices in the network is maintained at a similar level.

To this end, REE-MAC uses two types of superframes that operate simultaneously in different frequency bands: WET superframe and WIT superframe. In the WET superframe, a power transmitting unit (PTU) serving as a central coordinator supplies power to power receiving units (PRUs) (i.e., sensor devices) using the TDMA scheme. In the WIT superframe, multiple PRUs compete to transmit data packets to the PTU using CSMA/CA. At the beginning of each superframe, the PTU estimates the residual energy of individual PRUs changed due to their energy consumption and energy harvesting during the previous superframe. The PTU then determines the PRUs’ charging priorities, based on which it allocates dedicated power slots (DPSs) within the WET superframe.

We performed an experimental simulation to verify the superiority of REE-MAC over FF-WPT [25] and HE-MAC [19], which are the representative MAC protocols for WPSNs of out-of-band and in-band WET approaches, respectively. The results demonstrated that REE-MAC achieves 18.08% and 145.60% higher average harvested energy, 81.03% and 64.21% shorter average freezing time, and 100.49% and 135.56% higher fairness than FF-WPT and HE-MAC, respectively.

The rest of this paper is organized as follows. In Section 2, we present a system model for REE-MAC. In Section 3, the detailed operation of REE-MAC is described. The simulation configuration and results are presented in Section 4. Finally, Section 5 concludes this paper.

## 2. System Model

**Figure 1:**
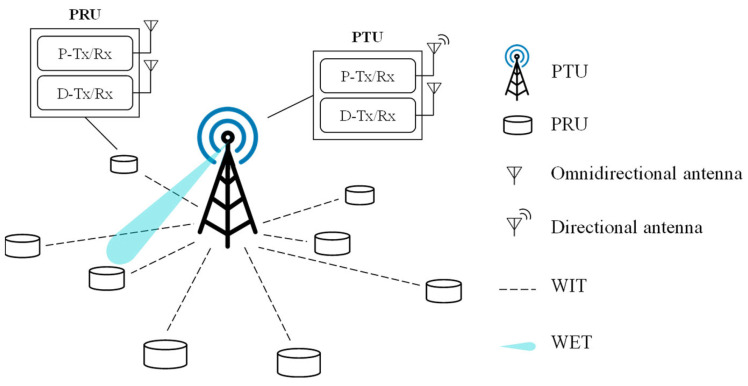
System architecture of REE-MAC.

Figure 1 illustrates the system architecture of the considered WPSN, which consists of a single PTU and multiple PRUs. The PRUs are deployed within the transmission range of the PTU. At the request of the PRU, the PTU either transfers power to the PRU or receives data from the PRU, for which the PTU and PRU are equipped with two types of transceivers: a power transceiver (P-Tx/Rx) for WET and a data transceiver (D-Tx/Rx) for WIT. The P-Tx/Rx and D-Tx/Rx in a device operate independently in different frequency bands and interact using internal signaling—enabling the WET and WIT of REE-MAC to be performed simultaneously.

Furthermore, the P-Tx/Rx and D-Tx/Rx of PTU always keep their radio on, while for the PRU, only its D-Tx/Rx is always on. The PRU keeps its P-Tx/Rx turned on to receive a beacon and power only in WET slots allocated to it and turns it off in other WET slots to prevent unnecessary energy consumption due to idle listening. Both the PTU and PRU have two antennas. The PTU has a directional antenna with a fixed beamwidth for WET and an omnidirectional antenna for WIT. We consider an adaptive array smart antennas as a directional antenna, which adjusts the beam direction by adapting the phase distribution of its antenna array elements. The PTU can transfer power to multiple PRUs using such a directional antenna. The PRU has two omnidirectional antennas for WET and WIT, respectively.

In REE-MAC, for determining when and how long to transfer power to individual PRUs, the PTU estimates the power that each PRU can receive per unit time, derived by Equation (1).

(1) $$P_{r,i}=P_{t}G_{t}G_{r}{\left(\frac{\lambda }{4\pi }\right)}^{2}d_{i}{}^{-\alpha },$$

where $P_{r,i}$ is the power that the *i*-th PRU receives per second and $P_{t}$ is the transmission power of the PRU. $G_{t}$ and $G_{r}$ are the antenna gain of the PTU and the PRU, respectively. We consider a flat-top radiation pattern as the directional antenna model of the PTU [27]; thus, $G_{t}$ is the same as 2π/θ, where θ is the beamwidth of the directional antenna of the PTU. In contrast, because the PRU receives power from the PTU using its omnidirectional antenna, $G_{r}$ is equal to one [28]. λ, $d_{i}$, and α refer to the wavelength, distance between the PTU and *i*-th PRU, and path loss exponent, respectively.

## 3. Design of REE-MAC

**Figure 2:**
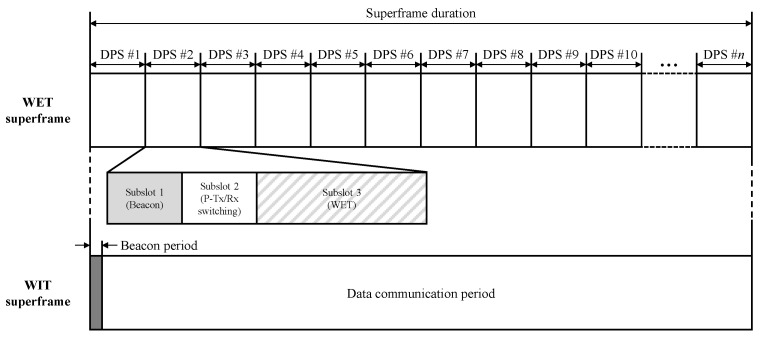
WET and WIT superframe structure.

Figure 2 illustrates a superframe structure of the REE-MAC. The REE-MAC maintains a dual superframe structure—WET and WIT superframes—operated in separated frequency bands. In the WET superframe, the PTU transfers power to the PRUs according to their dedicated schedule using the TDMA scheme. The PRUs use the harvested energy to exchange control messages and data packets using contention-based CSMA/CA in the WIT superframe. The WET superframe is divided into multiple equally sized DPSs, each allocated to an individual PRU for its exclusive power reception.

The DPS consists of three subslots: a beacon subslot, a P-Tx/Rx switching subslot, and a WET subslot. In the beacon subslot, the PTU or PRU beacon is broadcasted. The PTU beacon is transmitted in the beacon subslot of the first DPS in the WET superframe, through which the PTU informs the PRUs of the allocation result for the DPSs in the WET superframe. In the beacon subslot of the remaining DPSs in the WET superframe, the PRUs request power transfer by transmitting a PRU beacon to the PTU. In the P-Tx/Rx switching subslot, the PTU obtains the direction of the PRU from the received PRU beacon, and the PRU switches the mode of its P-Tx/Rx from Tx to Rx for harvesting the energy.

The direction of the PRU indicates the phase of the PRU beacon signal transmitted from the PRU, through which the PTU can change the beam direction of its antenna toward the corresponding PRU to transfer power to it. In the WET subslot, the PTU transfers power to the allocated PRU. The WIT superframe consists of a beacon period and a data communication period. In the beacon period in the WIT superframe, the PTU broadcasts a beacon including the WIT superframe parameters through D-Tx/Rx. The PTU and PRUs compete freely with each other for channel access using the CSMA/CA scheme.

**Figure 3:**
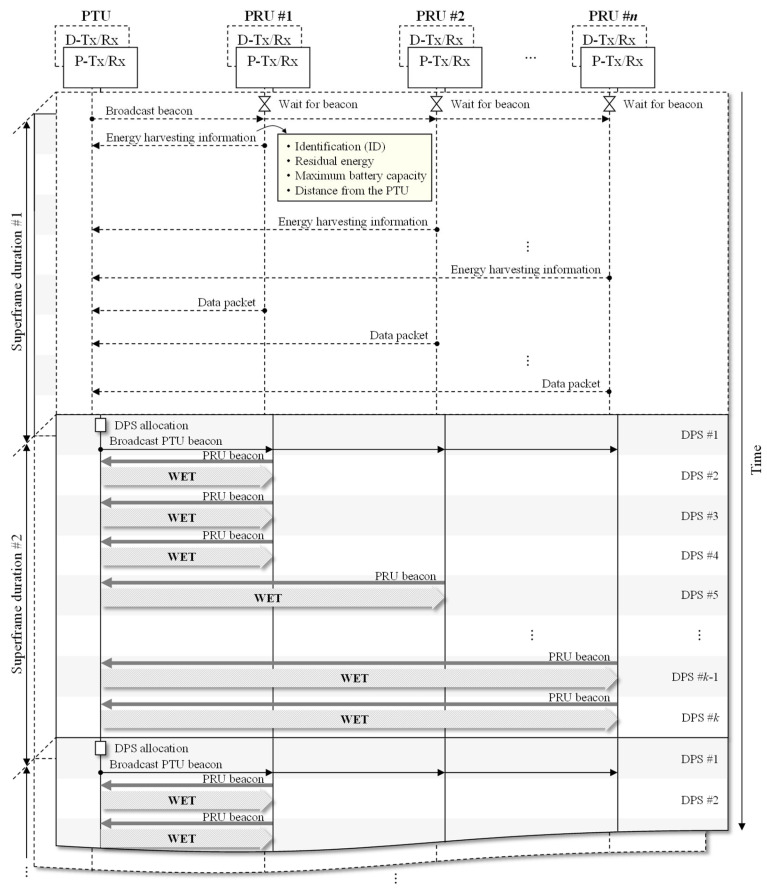
Overall operation of REE-MAC.

Figure 3 illustrates the overall operation of the REE-MAC. The solid and dashed lines represent the operation of the devices in WET and WIT superframes, respectively. These two superframes are expressed to overlap each other because the WET and WIT operations are performed simultaneously in different frequency bands. At the beginning of the WIT superframe, the PTU first broadcasts a beacon using D-Tx/Rx. Upon receiving the beacon from the PTU, the PRU transmits energy harvesting information, such as the node identifier (ID), residual energy, maximum battery capacity, and distance from the PTU, to the PTU.

This transmission of energy harvesting information for each PRU is performed only once throughout the entire network lifetime. The PTU and PRUs then exchange control messages and data packets using CSMA/CA. At the beginning of the WET superframe, the PTU performs the DPS allocation, which consists of three operations: calculating the number of DPSs required for individual PRUs to be fully charged, calculating the number of DPSs to be allocated to individual PRUs, and determining the charging priority for individual PRUs.

After the DPS allocation is completed, the PTU broadcasts the PTU beacon to notify PRUs of the scheduled WET superframe structure in the beacon subslot of the first DPS in the WET superframe. Then, in the beacon subslot in the subsequent DPSs allocated to individual PRUs, the PRU sends its PRU beacon to the PTU. With the PRU beacon, the PTU acquires the direction of the PRU and transfers power to the corresponding PRU in the WET subslot.

In estimating the residual energy of individual PRUs, the PTU first calculates the number of beacon, successful, collided, and idle slots in the previous WIT superframe. The beacon slot indicates a slot used by the PTU and PRU to transmit and receive the beacon. The successful slot indicates a slot used for successful transmission between the PTU and PRU. The collided slot indicates a slot in which collision occurs due to the data packets simultaneously transmitted from two or more PRUs. The idle slot is one in which no PRUs have transmitted the data packets due to the random backoff. It is assumed that *n* PRUs in the network always have the data packets to transmit in a saturation condition. The PTU maintains the node ID of PRUs (i.e., **ID**), the distance from itself to each PRU obtained from the received PRU beacons (i.e., **D**), the neighbor’s node ID of each PRU (i.e., **Nbr**_(*i*)_), and the number of data packets received from each PRU during the previous superframe (i.e., **Npkt**), which are represented by the matrices, as in Equation (2).

(2) $$ID=\left[id_{\left(1\right)},\;id_{\left(2\right)},\;\cdots ,id_{\left(i\right)},\;\cdots ,\;id_{\left(n\right)}\right],\;\;\;0<i\leq n\;D=\left[d_{\left(1\right)},\;d_{\left(2\right)},\;\cdots ,\;\;d_{\left(i\right)},\;\cdots ,d_{\left(n\right)}\right],\;\;\;0<i\leq n\;Nbr_{\left(i\right)}=\left[id_{\left(i,1\right)},\;id_{\left(i,2\right)},\;\cdots ,\;id_{\left(i,l\right)}\right]\;,\;\;\;0<i\leq n,\;\;\;0\leq l\leq (n-1)\;Npkt=\left[npkt_{\left(1\right)},\;npkt_{\left(2\right)},\;\cdots ,npkt_{\left(i\right)},\cdots ,\;npkt_{\left(n\right)}\right],\;\;\;0<i\leq n,$$

where $id_{\left(i\right)}$ is the node ID for the *i*-th PRU, *n* is the number of PRUs in the network, $d_{\left(i\right)}$ is the distance from the PTU to the *i*-th PRU, $id_{\left(i,l\right)}$ is the node ID of the *l*-th neighbor PRU of the *i*-th PRU, and $npkt_{\left(i\right)}$ is the number of data packets received from the *i*-th PRU during the previous WIT superframe.

The number of beacon and successful slots in the previous WIT superframe can be calculated using $L_{beacon}$ and **Npkt**, where $L_{beacon}$ is the length of the beacon subslot of DPS. We calculate the number of collided and idle slots by considering the collision and idle probabilities in the remaining slots except for beacon and successful slots in the previous WIT superframe as $p_{col}$ and $p_{idle}$, respectively [29]. $p_{col}$ is the probability that two or more PRUs transmit the data packet in a randomly chosen slot excluding beacon and successful slots in the previous WIT superframe, as defined by Equation (3).

(3) $$p_{col}=\sum_{k=2}^{n}\left(\begin{array}{c} n\\ k \end{array}\right)\;\tau^{k}{\left(1-\tau \right)}^{n-k},$$

where τ is the probability that the PRU transmits a data packet in a randomly chosen slot excluding beacon and successful slots in the previous WIT superframe. $p_{idle}$ is the probability that no PRU transmits a data packet in a randomly chosen slot, excluding beacon and successful slots in the previous WIT superframe, as defined by Equation (4).

(4) $$p_{idle}=1-p_{col}$$

**Figure 4:**
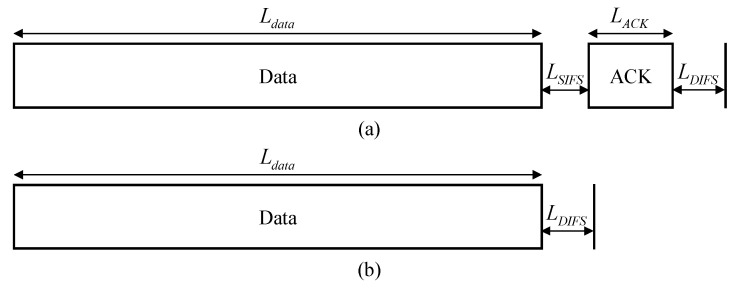
Timing diagram: (**a**) successful transmission, (**b**) collision.

The total number of slots in the previous WIT superframe, $n_{totalSlots}$, can then be calculated by Equation (5).

(5) $$n_{totalSlots}=L_{dataSF}/L_{BP},$$

where $L_{BP}$ is the slot length, which is the same as a unit backoff period. The numbers of beacon, successful, collided, and idle slots in the previous WIT superframe, $n_{beaconSlots}$, $n_{succSlots}$, $n_{colSlots}$, and $n_{idleSlots}$, can be calculated by Equations (6)–(9), respectively.

(6) $$n_{beaconSlots}=L_{beacon}/L_{BP}$$

(7) $$n_{succSlots}=\left(L_{succ}\sum_{i=1}^{n}n_{pkt,i}\right)/L_{BP}$$

(8) $$n_{colSlots}=p_{col}\left(n_{totalSlots}-n_{beaconSlots}-n_{succSlots}\right)$$

(9) $$n_{idleSlots}=p_{idle}\left(n_{totalSlots}-n_{beaconSlots}-n_{succSlots}\right),$$

where $L_{succ}$ is the time taken for successful transmission between the PTU and PRUs. Figure 4 illustrates the timing diagrams for the successful transmission and collision. $L_{data}$, $L_{SIFS}$, $L_{ACK}$, and $L_{DIFS}$ are the length of a data packet, a short inter-frame space (SIFS), an acknowledgment (ACK), and a distributed inter-frame space (DIFS), respectively.

The PTU then calculates $E_{beacon,i}$, $E_{succ,i}$, $E_{col,i}$, and $E_{idle,i}$ which are the amounts of energy consumed by the *i*-th PRU in the beacon, successful, collided, and idle slots in the previous WIT superframe, respectively, considering the timing diagrams. $E_{beacon,i}$ is the energy consumed by the *i*-th PRU to receive the beacon, as defined by Equation (10).

(10) $$E_{beacon,i}=n_{beaconSlots}E_{rx}L_{BP},$$

where $E_{rx}$ is the energy consumed per second by a PRU when its D-Tx/Rx is in the Rx state. $E_{succ,i}$ is the energy consumed by the *i*-th PRU during successful slots in the previous WIT superframe, as defined by Equation (11).

(11) $$E_{succ,i}=E_{succTx,i}+E_{succRx,i}+E_{succIdle,i},$$

where $E_{succTx,i}$ is the energy consumed by the successful transmission of the *i*-th PRU when its D-Tx/Rx is in the Tx state. $E_{succRx,i}$ is the energy consumed by the successful transmission of neighbors of the *i*-th PRU when its D-Tx/Rx is in the Rx state. Specifically, $E_{succRx,i}$ is the energy consumption of the *i*-th PRU in successful slots when it overhears the data packets transmitted by other PRUs in the previous WIT superframe.

Furthermore, $E_{succIdle,i}$ is the energy consumed by the successful transmission of the PRUs not adjacent to the *i*-th PRU when the D-Tx/Rx is in an idle state. $E_{succTx,i}$ can be calculated by Equation (12).

(12) $$E_{succTx,i}=n_{pkt,i}\left(E_{tx}L_{data}+E_{rx}L_{ACK}+E_{idle}\left(L_{SIFS}+L_{DIFS}\right)\right),$$

where $E_{tx}$ and $E_{idle}$ are the energy consumed per second by the PRU when its D-Tx/Rx is in the Tx and idle states, respectively. $E_{succRx,i}$ can be calculated by Equation (13).

(13) $$E_{succRx,i}=n_{succNbr,i}\left(E_{rx}L_{data}+E_{idle}L_{ACK}+E_{idle}\left(L_{SIFS}+L_{DIFS}\right)\right),$$

where $n_{succNbr,i}$ is the total number of data packets successfully transmitted to the PTU by neighbor PRUs of the *i*-th PRU. $E_{succIdle,i}$ can be calculated by Equation (14).

(14) $$E_{succIdle,i}=\left(n_{succSlots}-\left(n_{pkt,i}L_{data}\right)-\left(n_{succNbr,i}L_{data}\right)\right)E_{idle}L_{BP}$$

$E_{col,i}$ is the energy consumed by the *i*-th PRU during collided slots in the previous WIT superframe, as defined by Equation (15).

(15) $$E_{col,i}=E_{colTx,i}+E_{colRx,i}+E_{colIdle,i},$$

where $E_{colTx,i}$ is the energy consumed by the data packet transmission of the *i*-th PRU when one or more other PRUs transmits the data packet simultaneously. $E_{colRx,i}$ is the energy consumed by the *i*-th PRU due to the collision caused by two or more PRUs including at least one neighbor. Specifically, $E_{colRx,i}$ is the energy consumption of the *i*-th PRU when it overhears the data packets transmitted by two or more PRUs, including at least one neighbor, simultaneously. $E_{colIdle,i}$ is the energy consumed by the *i*-th PRU when two or more PRUs, excluding itself and its neighbors, transmit the data packet simultaneously. In this case, two or more PRUs outside the transmission range of the *i*-th PRU transmit the data packet simultaneously. $E_{colTx,i}$, $E_{colRx,i}$, and $E_{colIdle,i}$ can be calculated by Equations (16)–(18), respectively.

(16) $$E_{colTx,i}=n_{colSlots}\left(E_{tx}L_{data}+E_{idle}L_{DIFS}\right)\left(\tau \sum_{k=1}^{n-1}\left(\begin{array}{c} n-1\\ k \end{array}\right)\tau^{k}{\left(1-\tau \right)}^{n-k-1}\right),\;\;\;1\leq k\leq (n-1)$$

(17) $$E_{colRx,i}=\left(n_{colSlots}\left(E_{rx}L_{data}+E_{idle}L_{DIFS}\right)\left(1-\tau \right)\sum_{k=2}^{n-1}\left(\begin{array}{c} n-1\\ k \end{array}\right)\tau^{k}{\left(1-\tau \right)}^{n-k-1}\right)-E_{colIdle,i},\;\;\;2\leq k\leq (n-1)$$

(18) $$E_{colIdle,i}=n_{colSlots}E_{idle}\left(\left(1-\tau \right){\left(1-\tau \right)}^{n_{nbr,i}}\sum_{k=2}^{n-n_{nbr,i}-1}\left(\begin{array}{c} n-n_{nbr,i}-1\\ k \end{array}\right)\tau^{k}\right),\;\;\;2\leq k\leq (n-n_{nbr,i}-1)$$

Finally, $E_{idle,i}$ is the energy consumed by the *i*-th PRU during the idle slots in the previous WIT superframe when its D-Tx/Rx is in an idle state, as defined by Equation (19).

(19) $$E_{idle,i}=n_{idleSlots}E_{idle}L_{BP}$$

For estimating the residual energy of the *i*-th PRU, the PTU must calculate not only the energy consumed by the *i*-th PRU but also the energy harvested by the *i*-th PRU (i.e., $E_{rx,i}$) in the previous WET superframe, as defined by Equation (20).

(20) $$E_{rx,i}=\sum_{k=1}^{n_{DPS,i}}E_{DPS,i},$$

where $n_{DPS,i}$ is the number of DPSs allocated to the *i*-th PRU in the previous WET superframe. $E_{DPS,i}$ is the energy harvested by the *i*-th PRU during one DPS, as defined by Equation (21).

(21) $$E_{DPS,i}=\eta P_{r,i}L_{WET},$$

where η is the energy harvesting efficiency of the PRU, $P_{r,i}$ is the power received per second of the *i*-th PRU (refer to Equation (1) in Section 2), and $L_{WET}$ is the length of the WET subslot.

Consequently, the residual energy of the *i*-th PRU (i.e., $E_{res,i}$) can be represented by Equation (22).

(22) $$E_{res,i}=E_{res,i}-E_{beacon,i}-E_{succ,i}-E_{col,i}-E_{idle,i}+E_{rx,i}$$

Based on the knowledge of the residual energy estimation for individual PRUs, the PTU performs the DPS allocation at the beginning of every WET superframe. Algorithm 1 presents the DPS allocation procedure, which consists of three operations: (1) the calculation of the number of DPSs required for individual PRUs to be fully charged, (2) the calculation of the number of DPSs to be allocated to individual PRUs, and (3) the determination of charging priority for individual PRUs.
**Algorithm 1.** DPS allocation1:**INITIALIZE** $N_{DPS}$ to [],$N_{sortedDPS}$ to [],$I_{DPS}$ to [],$I_{startDPS}$ to [],$S_{DPS}$ to 0,      $n_{reqDPS,i}$ to 0, cnt to 2, $n_{avaDPS}$ to $n_{totalDPS}-1$, $E_{\max,i}$ to 12:/* Calculation of the number of DPSs required for individual PRUs to be fully charged */3:**FOR** each PRU, i, i∈[1,n]
4:  $n_{reqDPS,i}\leftarrow (E_{\max,i}-E_{res,i})/E_{DPS,i}$
5: 
$S_{DPS}\leftarrow S_{DPS}+n_{reqDPS,i}$
6:**ENDFOR**7:/* Calculation of the number of DPSs to be allocated to individual PRUs */8:**FOR** each PRU, i, i∈[1,n]
9: 
$n_{DPS,i}\leftarrow round(n_{avaDPS}(n_{reqDPS,i}/S_{DPS}))$
10: 
$N_{DPS}[i]\leftarrow n_{DPS,i}$
11:**ENDFOR**12:/* Determination of the charging priority for individual PRUs */13:$\left[N_{sortedDPS},I_{DPS}]\leftarrow sort(N_{DPS}{,}^{\prime }descend^{\prime }\right)$14:**FOR** each PRU, i, i∈[1,n]
15: 
$I_{startDPS}[I_{DPS}[i]]\leftarrow cnt$
16: 
$cnt\leftarrow cnt+N_{sortedDPS}[i]$
17:**ENDFOR**18:**RETURN** $N_{DPS}$ and $I_{startDPS}$


In the algorithm, the PTU initializes the attributes and variables (i.e., $I_{DPS}$, $I_{startDPS}$, $N_{DPS}$, $N_{sortedDPS}$, $S_{DPS}$, $n_{reqDPS,i}$, *cnt*, $n_{avaDPS}$, and $E_{\max,i}$), where $I_{DPS}$ and $I_{startDPS}$ are the attributes to store the indices of the PRUs, $N_{DPS}$ and $N_{sortedDPS}$ are the attributes to track the number of DPSs allocated to them, and $S_{DPS}$, $n_{reqDPS,i}$, *cnt*, $n_{avaDPS}$, and $E_{\max,i}$ are the variables for counting the number of DPSs. $I_{DPS}$ includes the indices of elements in $N_{DPS}$ before $N_{DPS}$ is sorted in descending order as $N_{sortedDPS}$. $I_{startDPS}$ includes the starting indices of DPSs allocated to individual PRU in the WET superframe. $N_{DPS}$ includes the numbers of DPSs allocated to PRUs. $N_{sortedDPS}$ is $N_{DPS}$ sorted in descending order according to the values of elements included in $N_{DPS}$. $I_{DPS}$, $I_{startDPS}$, $N_{DPS}$, and $N_{sortedDPS}$ are represented by one-dimensional arrays. $S_{DPS}$ is the sum of the number of DPSs required by PRUs, $n_{reqDPS,i}$, which is the number of DPSs required for the *i*-th PRU to be fully charged from the current residual energy to the maximum battery capacity. *cnt* is a counter value used to calculate the starting index of DPSs allocated to individual PRU in the WET superframe. $n_{avaDPS}$ is the number of available DPSs in the WET superframe excluding the first DPS and DPSs already allocated to PRUs, initialized to $n_{totalDPS}-1$. $n_{totalDPS}$ is the total number of DPSs in the WET superframe.

In the first operation, the PTU obtains the number of DPSs required for individual PRUs to be fully charged (i.e., $n_{reqDPS,i}$), calculated using the energy required for each PRU’s battery to be charged to its maximum battery capacity (i.e., $E_{\max,i}-E_{res,i}$) and the energy harvested during one DPS (i.e., $E_{DPS,i}$) (line 4). The PTU then calculates the sum of the number of DPSs required by all PRUs (i.e., $S_{DPS}$) (line 5).

In the second operation, the PTU obtains the number of DPSs to be allocated to individual PRUs (i.e., $n_{DPS,i}$) according to the ratio of the number of DPSs required by each PRU to the sum of the number of DPSs required by all PRUs (i.e., $n_{reqDPS,i}/S_{DPS}$) (line 9). Accordingly, the PTU maintains the list of the number of DPSs to be allocated to individual PRUs (i.e., $N_{DPS}[i]$) (line 10).

Finally, the PTU determines the charging priority for individual PRUs by sorting $N_{DPS}[i]$ in descending order according to the number of DPSs (line 13). The starting index of the DPSs in the WET superframe allocated to each PRU is calculated according to the charging priority for individual PRUs (line 15). Consequently, the PRU with low residual energy can perform the energy harvesting ahead of other PRUs.

After completing the DPS allocation algorithm, the PTU includes the number of DPSs (i.e., $N_{DPS}$) and the starting index (i.e., $I_{startDPS}$) of DPSs allocated to PRUs in the PTU beacon and broadcasts the PTU beacon. Based on the results of DPS scheduling, the PTU transfers power to the PRUs, and the PRUs perform energy harvesting.

## 4. Performance Evaluation

We evaluated the performance of REE-MAC using experimental simulations with the MATLAB simulator. The simulation results were compared with those of FF-WPT [25] and HE-MAC [19]. FF-WPT is an out-of-band solution that transfers power to devices using a different frequency band separated from that used to transmit data packets. In contrast, HE-MAC is an in-band solution that performs both power transfer and data transmission within the same frequency band. In the following subsections, we present in detail the simulation setup and configuration and the simulation results.

### 4.1. Simulation Configuration

In the simulation, we considered a WPSN consisting of one PTU and multiple PRUs. We assumed that each PRU is randomly deployed within the communication range of the PTU, set to 4 m. We further assumed that the PRU always has the data packets to transmit to the PTU. In the simulation, the number of PRUs varies from 2 to 20. The performance of REE-MAC was compared with those of FF-WPT [25] and HE-MAC [19] in terms of average harvested energy, average consumed energy, average freezing time, residual energy distribution, throughput distribution, and fairness indices for residual energy and throughput.

We investigated fairness by considering the freezing state, which indicates a state of the device in which the PRU cannot transmit the data packets due to a lack of residual energy. In the simulation, the PRU enters the freezing state when the residual energy is less than a pre-configured energy threshold. Then, if it exceeds a pre-configured active threshold, it is released from the freezing state. The active threshold indicates the minimum residual energy required for the PRU to resume the data packet transmission in the freezing state. It is set larger than the energy threshold to prevent the PRUs from entering the freezing state again immediately after they are released from the freezing state.

In FF-WPT, the WET operation is performed according to TDMA-based round-robin scheduling, while the WIT operation is performed using the CSMA/CA scheme. In FF-WPT, the interval required for exchanging control messages was set to 300 µs. Furthermore, the number of energy frames of each PRU is determined considering packet size and harvested energy per second. In HE-MAC, the arbitration inter-frame spaces (AIFSs) for PTU and PRU (i.e., $AIFS_{PTU}$ and $AIFS_{PRU}$) were set to 50 and 70 µs, respectively. The simulation was iterated 50 times. The detailed simulation parameters are listed in Table 1.

### 4.2. Simulation Results

**Figure 5:**
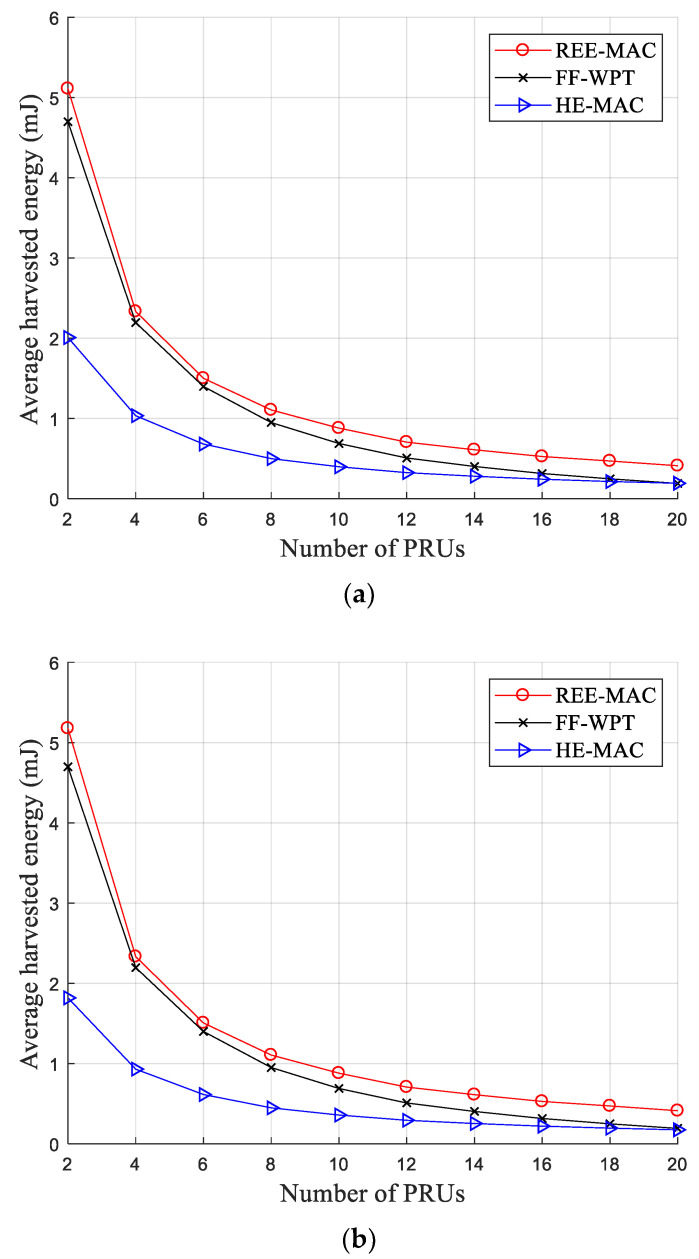
Average harvested energy: (**a**) packet size of 100 bytes; (**b**) packet size of 200 bytes.

Figure 5a,b illustrate the variations in the average harvested energy for 100- and 200-byte packets, respectively. The average harvested energy decreases as the number of PRUs in the network increases due to decreased energy harvested by individual PRUs per WET superframe. In both figures, REE-MAC exhibits a higher average harvested energy compared with both FF-WPT and HE-MAC. Regarding the DPS allocation operation, REE-MAC requires a shorter time overhead than FF-WPT. In REE-MAC, the PTU performs DPS allocation by estimating the residual energy for individual PRUs and informs the PRUs of the number of allocated DPSs through a PTU beacon at the beginning of the WET superframe. Therefore, REE-MAC entails only time overhead for sending one PTU beacon.

In contrast, FF-WPT incurs a long time overhead because, in FF-WPT, the PTU needs to exchange control messages with all PRUs to obtain the distance between itself and each PRU. Furthermore, HE-MAC exhibits the lowest average harvested energy because, in HE-MAC, the exchange of control messages, the transmission of data packets, and energy harvesting are all performed in the same frequency band.

As presented in Table 2, the average harvested energy of REE-MAC and FF-WPT are almost identical, even if the packet size increases from 100 to 200 bytes, because the packet size does not affect the average harvested energy of PRUs in out-of-band approaches. However, in HE-MAC, the average harvested energy decreases slightly when the packet size increases from 100 to 200 bytes because the proportion of the energy harvesting time within the WET superframe is reduced. Quantitatively, the average harvested energy of REE-MAC is 17.79% and 18.38% higher than that of FF-WPT when the PRUs transmit the 100- and 200-byte packets, respectively. Moreover, the average harvested energy of REE-MAC is 132.15% and 159.04% higher than that of HE-MAC when the PRUs transmit the 100- and 200-byte packets, respectively.

**Figure 6:**
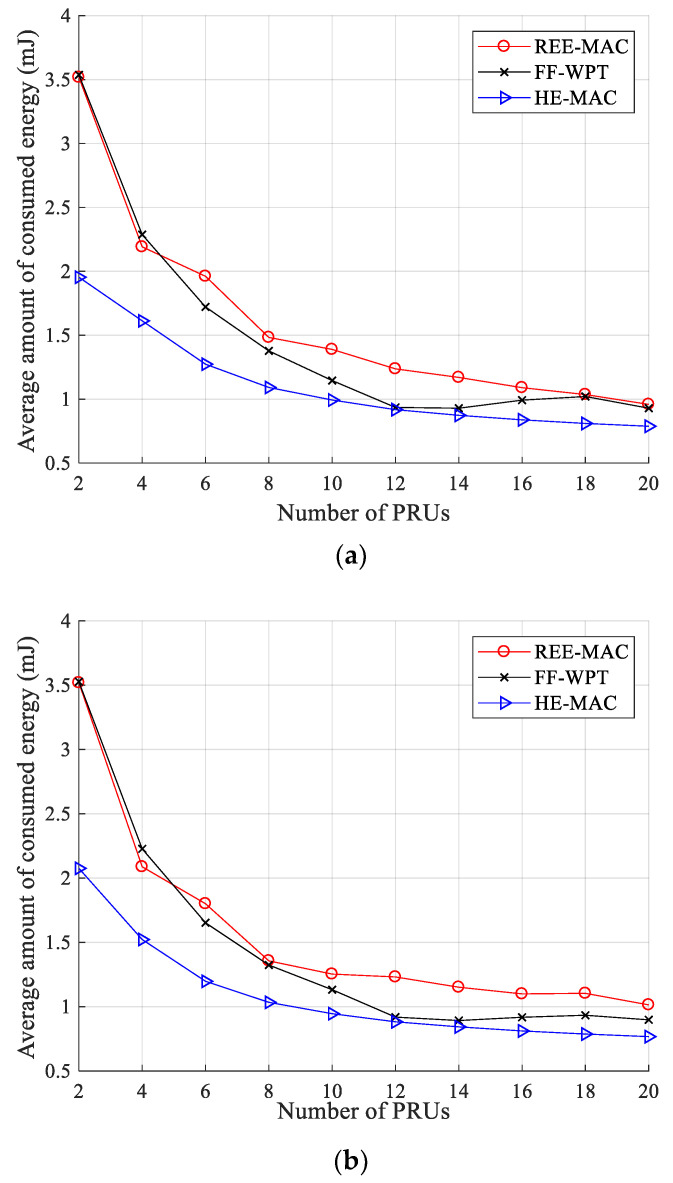
Average consumed energy: (**a**) packet size of 100 bytes; (**b**) packet size of 200 bytes.

Figure 6a,b illustrate the variations in the average consumed energy for 100- and 200-byte packets, respectively. In REE-MAC, the average consumed energy decreases as the number of PRUs increases because the number of data packets transmitted by PRUs gradually decreases due to the increase in collisions and backoff delay. Consequently, the number of transmissions of PRUs is reduced, reducing the energy consumed by PRUs. In FF-WPT, as in REE-MAC, the average consumed energy tends to decrease overall as the number of PRUs increases.

Furthermore, FF-WPT exhibits an average consumed energy similar to REE-MAC. However, in some sections (i.e., when the number of PRUs is 14 to 18), the average consumed energy of FF-WPT slightly increases. As the number of PRUs increases, the energy each PRU can harvest decreases, and accordingly, the number of PRUs entering the freezing state increases. Therefore, a relatively small number of PRUs transmit data packets to the PTU, and the average consumed energy can increase due to the reduced contention level.

HE-MAC exhibits lower average consumed energy compared with both REE-MAC and FF-WPT. In HE-MAC, the PRUs have relatively few transmission opportunities for data packets because both WET and WIT operations are performed within the same frequency band. Therefore, a small number of data packet transmissions reduces the energy consumed by the PRU. Quantitatively, when the PRUs transmit 100- and 200-byte packets, the average consumed energy of REE-MAC is 7.79% and 8.29% higher than that of FF-WPT, respectively. Moreover, it is 43.78% and 43.74% higher compared with HE-MAC, respectively.

**Figure 7:**
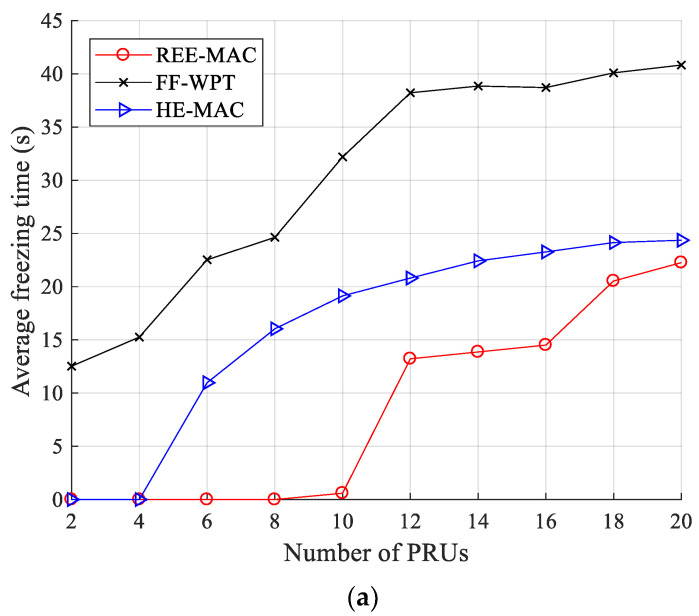

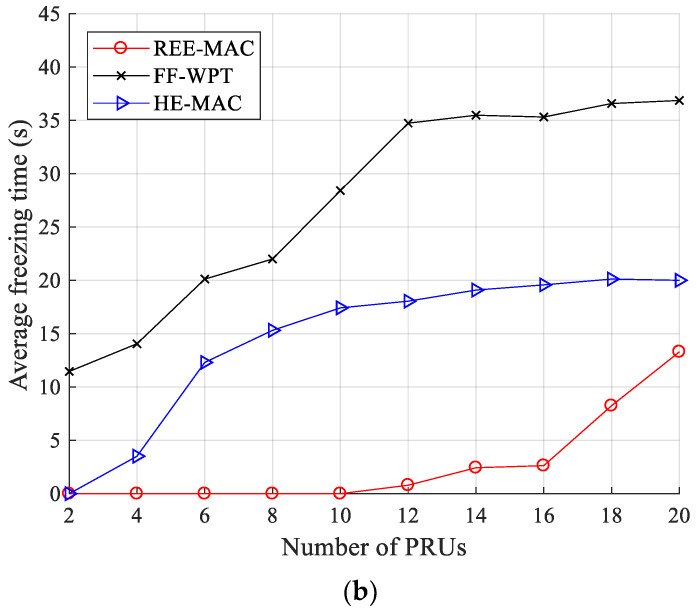
Average freezing time: (**a**) packet size of 100 bytes; (**b**) packet size of 200 bytes.

Figure 7a,b illustrate the variations in the average freezing time for 100- and 200-byte packets, respectively. The freezing time is the time the PRU is in the freezing state. As the number of PRUs increases, the energy harvested by each PRU decreases, and accordingly, the average freezing time of PRUs is highly likely to increase. REE-MAC exhibits a shorter average freezing time than both FF-WPT and HE-MAC. In REE-MAC, the DPS allocation is performed considering the residual energy of individual PRUs. Accordingly, in REE-MAC, PRUs with less residual energy are allocated more DPSs within the superframe. Therefore, all PRUs in the network maintain similar residual energy, and the time they are in the freezing state is relatively short.

When the sizes of data packets transmitted by PRUs are 100 and 200 bytes, the average freezing time of REE-MAC is almost zero until the numbers of PRUs are 10 and 12, respectively. However, if the numbers of PRUs exceed 10 and 12, the average freezing time of REE-MAC increases as the number of PRUs increases. FF-WPT consistently exhibits a longer average freezing time than both REE-MAC and HE-MAC because, in FF-WPT, the PTU transfers power to PRUs according to the distance without considering the residual energy of PRUs. Furthermore, individual PRUs harvest smaller energy due to the exchange of control messages required for WET operation. Accordingly, in FF-WPT, PRUs enter the freezing state more frequently.

When the number of PRUs is more than 12, the average freezing time of FF-WPT increases gradually. When the number of PRUs is increased to more than 12, the PRUs enter the freezing state more quickly due to the decrease in harvested energy. Therefore, it takes longer for the residual energy of PRUs in the freezing state to reach the active threshold.

In HE-MAC, the PRU occupying the channel uses the harvest-then-transmit scheme. Therefore, the PRU first harvests the energy required to transmit the data packet and then uses it to transmit the data packet. Other PRUs maintain an idle state to minimize energy consumption. Consequently, the average freezing time of HE-MAC is shorter than that of FF-WPT. Quantitatively, when the PRUs transmit 100- and 200-byte packets, the average freezing time of REE-MAC is 72.03% and 90.04% shorter than that of FF-WPT, respectively. It is also 47.26% and 81.15% shorter than that of HE-MAC.

**Figure 8:**
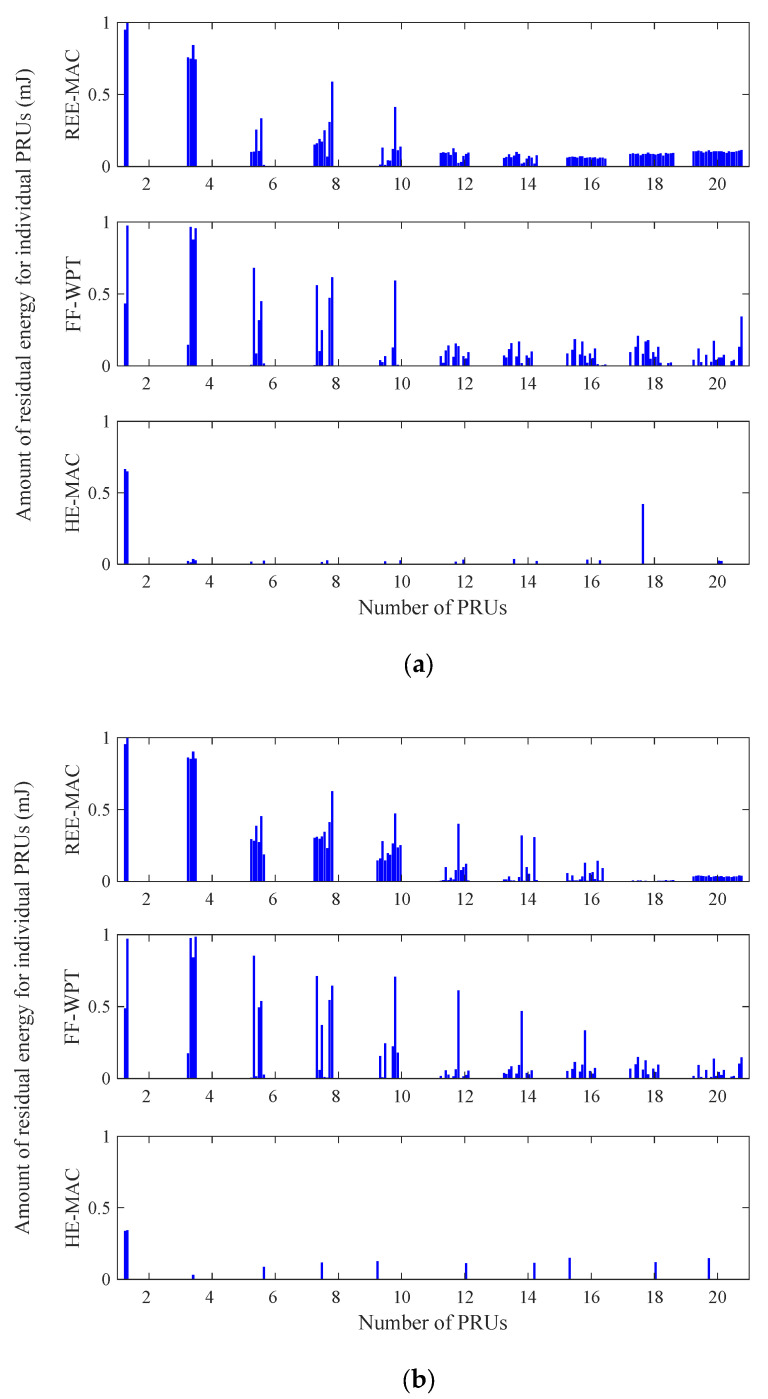
Residual energy distribution of individual PRUs: (**a**) packet size of 100 bytes; (**b**) packet size of 200 bytes.

Figure 8a,b illustrate the variations in the residual energy distribution of individual PRUs for 100- and 200-byte packets, respectively. Both figures indicate the residual energy distribution of individual PRUs in a specific round of the experimental simulation. In REE-MAC and FF-WPT, when the packet size increases, the residual energy of individual PRUs decreases due to an increase in the consumed energy.

In HE-MAC, the number of dead PRUs increases. A dead PRU indicates a PRU with a residual energy of zero. In REE-MAC, the difference between the residual energy of individual PRUs is slight compared with both FF-WPT and HE-MAC. REE-MAC enables PRUs to maintain similar residual energy through the DPS allocation considering the residual energy. In FF-WPT, the fluctuation in the residual energy distribution of individual PRUs is larger than that of REE-MAC because the DPSs are allocated considering only the distance between the PTU and individual PRUs. In HE-MAC, the residual energy of individual PRUs is almost zero except when the number of PRUs is 2 because the PRUs consume additional energy in addition to data packet transmission, and the residual energy of the PRUs gradually decreases. Consequently, in HE-MAC, more dead PRUs occur compared with REE-MAC and FF-WPT.

**Figure 9:**
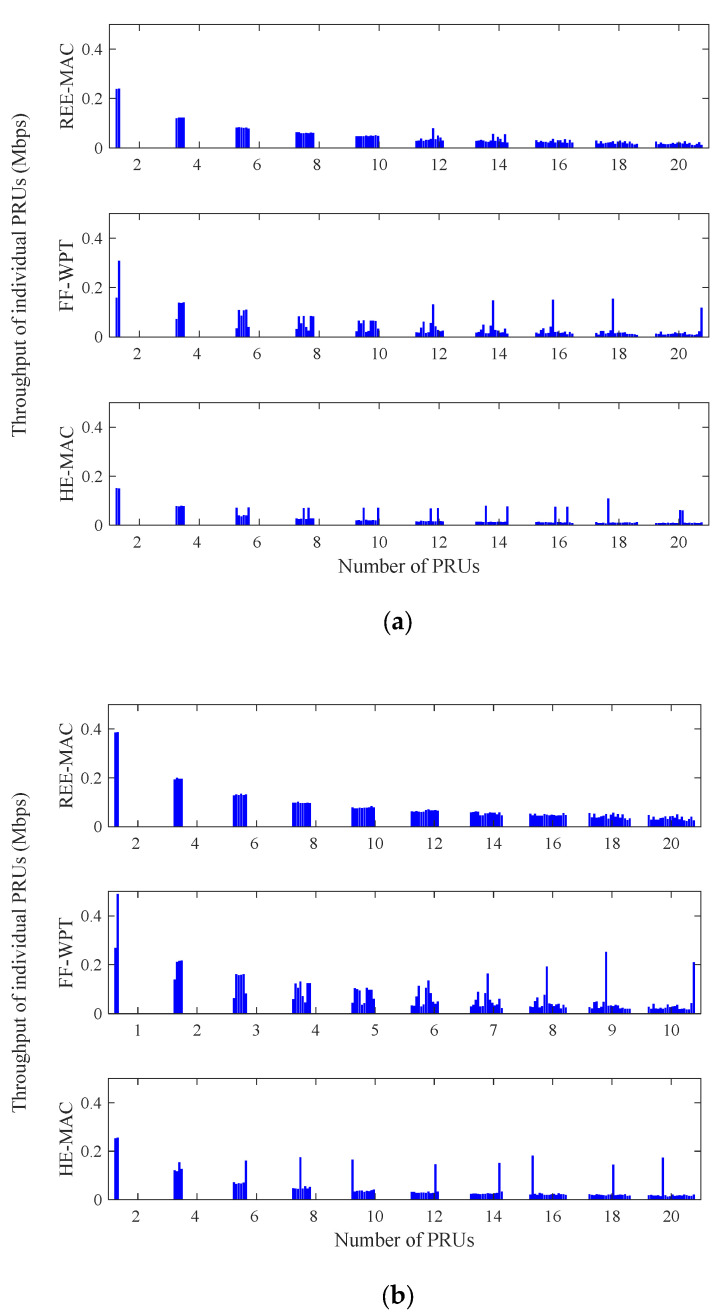
Throughput distribution of individual PRUs: (**a**) packet size of 100 bytes; (**b**) packet size of 200 bytes.

Figure 9a,b illustrate the variations in the throughput distribution of individual PRUs for 100- and 200-byte packets, respectively. Both figures indicate the throughput distribution of individual PRUs in a specific round of the experimental simulation. The fluctuation of the throughput distribution increases as the number of PRUs in the network increases. Moreover, when the packet size increases, the throughput of individual PRUs increases due to the decrease in the backoff delay. REE-MAC exhibits a constant throughput distribution regardless of the number of PRUs, compared with FF-WPT and HE-MAC. In REE-MAC, all PRUs in the network maintain similar throughput performance.

From the results of Figure 7 and Figure 8, in REE-MAC, the PRUs maintain the shortest freezing time, on average, and dead PRUs with zero residual energy rarely occur. Therefore, compared with FF-WPT and HE-MAC, the PRUs using REE-MAC can have relatively even transmission opportunities. However, in FF-WPT and HE-MAC, the transmission opportunity is biased toward some PRUs as the number of PRUs in the network increases. Accordingly, in FF-WPT and HE-MAC, the throughput distribution for specific PRUs becomes severely concentrated.

**Figure 10:**
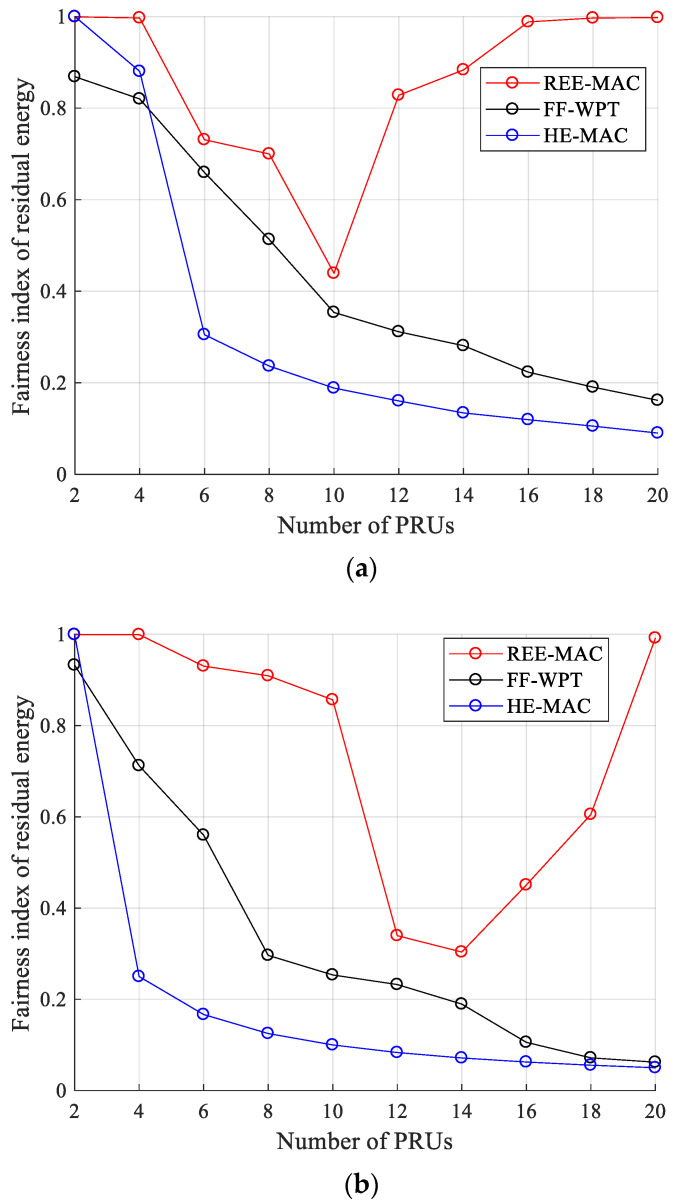
Fairness index for residual energy: (**a**) packet size of 100 bytes; (**b**) packet size of 200 bytes.

Figure 10a,b illustrate the fairness index for residual energy for 100- and 200-byte packets, respectively. The fairness index for residual energy ($F_{res}$) can be calculated by Equation (23) [30].

(23) $$F_{res}=\frac{\left(\sum_{i}^{n}x_{i}^{2}\right)}{n\cdot \sum_{i=1}^{n}x_{i}^{2}},$$

where n is the number of PRUs and $x_{i}$ is the fairness parameter, which represents the residual energy of the *i*-th PRU.

In REE-MAC, the fairness index for residual energy decreases and then increases again as the number of PRUs increases. For 100- and 200-byte packets, when the number of PRUs is 10 and 14 or less, respectively, the fairness index for residual energy of REE-MAC decreases as the number of PRUs increases. As the number of PRUs in the network increases, the difference in the residual energy between individual PRUs increases due to a decrease in the energy harvested by individual PRUs and the difference in the energy consumed by individual PRUs (refer to Figure 8).

In contrast, when the numbers of PRUs are larger than 10 and 14, respectively, the fairness index for residual energy of REE-MAC increases as the number of PRUs increases. As the number of PRUs increases, the energy harvested by individual PRUs decreases, and the number of PRUs in the freezing state increases accordingly. PRUs in the freezing state perform only WET operations until their residual energy reaches the active threshold. Therefore, as the residual energy of many PRUs in the freezing state approaches the active threshold, the fairness index for residual energy increases.

However, in FF-WPT and HE-MAC, the fairness index for residual energy decreases as the number of PRUs in the network increases. As depicted in Figure 7, the gap in transmission opportunities between PRUs increases as the average freezing time of PRUs increases. This gap increases the difference between the energy consumed by individual PRUs. Therefore, the fairness index for residual energy gradually decreases as the difference between the residual energy of individual PRUs increases. Quantitatively, when the PRUs transmit 100- and 200-byte packets, the fairness index for residual energy of REE-MAC is 95.34% and 116.23% higher than that of FF-WPT, respectively. It is also 165.88% and 276.00% higher compared with that of HE-MAC, respectively.

**Figure 11:**
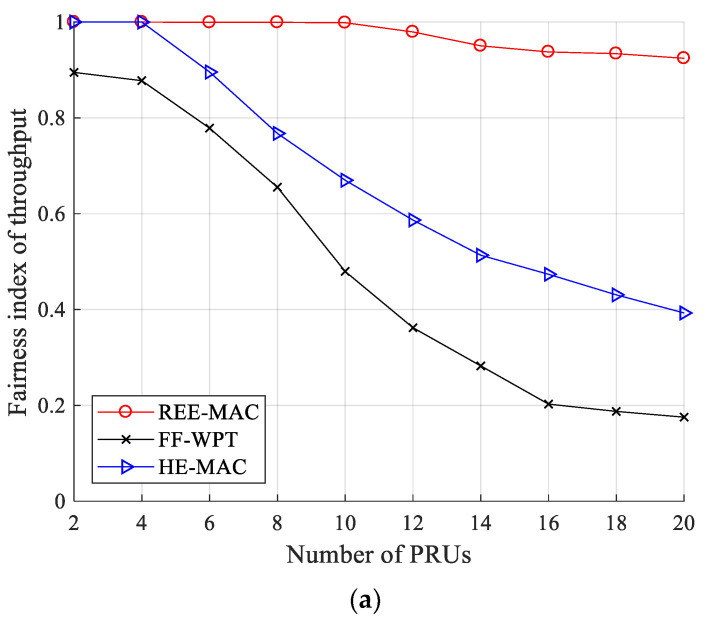

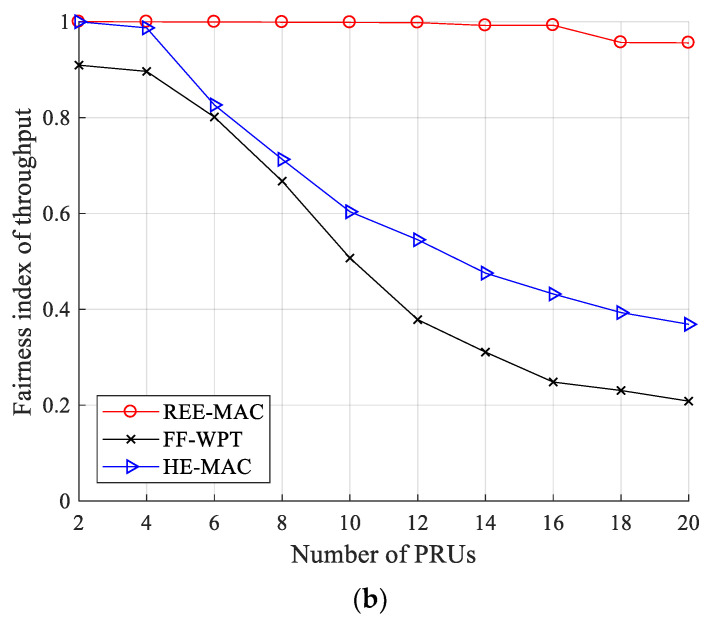
Fairness index for throughput: (**a**) packet size of 100 bytes; (**b**) packet size of 200 bytes.

Figure 11a,b illustrate the fairness index for throughput for 100- and 200-byte packets, respectively. The fairness index for throughput ($F_{th}$) can be calculated by Equation (24).

(24) $$F_{th}=\frac{\left(\sum_{i}^{n}y_{i}^{2}\right)}{n\cdot \sum_{i=1}^{n}y_{i}^{2}},$$

where n is the number of PRUs and $y_{i}$ is the fairness parameter indicating the number of data packets that the *i*-th PRU transmits to the PTU.

In each case of 100- and 200-byte packets, REE-MAC maintains the fairness index for throughputs of 0.924 and 0.956 or higher, respectively, regardless of the number of PRUs. As the number of PRUs increases, the energy harvested by individual PRUs decreases and the difference in energy consumed by individual PRUs increases. Nevertheless, in REE-MAC, the residual energy of PRUs is maintained similar to each other due to DPS allocation considering the residual energy of individual PRUs. Accordingly, individual PRUs achieve a high fairness index for throughput by having a similar transmission opportunity.

In contrast, FF-WPT exhibits a lower fairness index for throughput compared with both REE-MAC and HE-MAC. From the results in Figure 7, in FF-WPT, PRUs maintain the longest freezing time on average. The difference in throughput performance between PRUs in FF-WPT becomes significant because the increase of the freezing time causes the transmission opportunities to be biased to some PRUs. The fairness index for throughput of FF-WPT decreases as the number of PRUs increases regardless of the packet size because the average freezing time of PRUs increases due to the decrease in the energy harvested by individual PRUs.

HE-MAC exhibits a higher fairness index for throughput than FF-WPT regardless of the packet size. In HE-MAC, PRUs that are not in the freezing state maintain similar residual energy through the harvest-then-transmit scheme, and thus they have a relatively similar transmission opportunity. Quantitatively, when the PRUs transmit 100- and 200-byte packets, the fairness index for throughput of REE-MAC is 98.58% and 91.80% higher than that of FF-WPT, respectively. It is also 44.46% and 55.91% higher compared with HE-MAC, respectively.

## 5. Conclusions

This paper presents the REE-MAC protocol for WPSNs, which aims to reduce overhead due to control messages for scheduling the WET operation and provide fairness for data transmission opportunities to the sensor devices. REE-MAC achieves low overhead by numerically estimating the residual energy of individual PRUs without exchanging control messages. Furthermore, in REE-MAC, the PTU allocates the DPSs inversely proportional to the residual energy of individual PRUs, so that all PRUs in the network maintain similar residual energy. Thereby, it minimizes the energy depletion of some PRUs and provides individual PRUs with a fair data transmission opportunity. At the beginning of each superframe, the PTU calculates the consumed and harvested energy of individual PRUs and then estimates their residual energy. It then performs the DPS allocation based on the results of the residual energy estimation.

We conducted an experimental simulation to evaluate the performance of REE-MAC under the environment of changing network size and packet size. The results demonstrate that REE-MAC uses the residual energy estimation to reduce unnecessary waste of bandwidth due to the exchange of control messages, increasing the energy harvested by individual PRUs. Moreover, REE-MAC prevents the DPSs from biased allocation to some PRUs, reducing the freezing time of the PRUs due to lack of energy.

These operations of REE-MAC give similar transmission opportunities to PRUs in the network, ensuring higher fairness compared with FF-WPT and HE-MAC in terms of residual energy and throughput. On average, REE-MAC achieves 18.08% and 145.60% higher energy harvested, 81.03% and 64.21% shorter average freezing time, 105.79% and 220.94% higher fairness index for residual energy, and 95.19% and 50.18% higher fairness index for throughput, compared with FF-WPT and HE-MAC, respectively.

## Figures and Tables

**Table 1:** Simulation parameters.

Parameter	Value	Parameter	Value
Number of PRUs	2–20	d	0–4 m
Data rate	2 Mbps	Maximum battery capacity	1 mJ
WET superframe	1 s	Initial residual energy	0.6 mJ
WIT superframe	1 s	Active threshold	0.6 mJ
DPS length	10 ms	Energy threshold	0.1 mJ
Number of DPSs	100	$CW_{\min}$	31
Beacon subslot	40 µs	$CW_{\max}$	1023
P-Tx/Rx switching subslot	10 µs	$G_{t}$	12
WET subslot	9950 µs	$G_{r}$	1
RTS	20 bytes	$P_{tx}$	31.47 mA
CTS	14 bytes	$P_{rx}$	26.94 mA
ACK	14 bytes	$P_{idle}$	0.00156 mA
SIFS	10 µs	$P_{t}$	3000 mW
DIFS	50 µs	α	2.7
Beacon	15 bytes	η	0.85
Packet size	100–200 bytes	Slot length	20 µs

**Table 2:** Average harvested energy (mJ).

Packet Size	Protocol	Number of PRUs									
		2	4	6	8	10	12	14	16	18	20
100 bytes	REE-MAC	2.65	1.56	1.03	0.76	0.61	0.52	0.48	0.41	0.36	0.31
	FF-WPT	2.57	1.47	0.95	0.67	0.51	0.41	0.36	0.30	0.25	0.21
	HE-MAC	1.31	0.72	0.48	0.35	0.28	0.24	0.20	0.18	0.16	0.14
200 bytes	REE-MAC	2.65	1.56	1.03	0.76	0.61	0.52	0.46	0.41	0.36	0.31
	FF-WPT	2.57	1.47	0.95	0.67	0.51	0.41	0.36	0.30	0.25	0.21
	HE-MAC	1.20	0.65	0.44	0.32	0.25	0.21	0.18	0.16	0.14	0.13

## Data Availability

No new data were created or analyzed in this study. Data sharing is not applicable to this article.
